# Polymeric Properties of Telomeric G-Quadruplex Multimers: Effects of Chemically Inert Crowders

**DOI:** 10.1021/acs.biomac.5c00176

**Published:** 2025-04-08

**Authors:** Deniz Mostarac, Mattia Trapella, Luca Bertini, Lucia Comez, Alessandro Paciaroni, Cristiano De Michele

**Affiliations:** † Department of Physics, University of Rome La Sapienza, 00185 Rome, Italy; ‡ Department of Physics and Geology, University of Perugia, 06123 Perugia, Italy; § CNR - Istituto Officina dei Materiali (IOM), 06123 Perugia, Italy

## Abstract

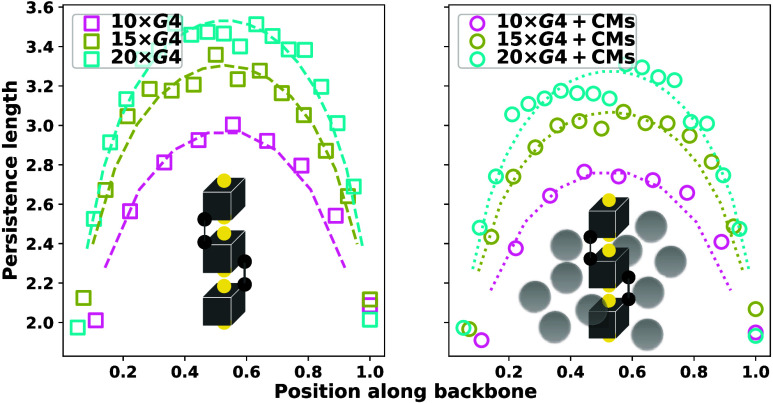

G-quadruplexes are noncanonical DNA structures rather ubiquitous in the human genome, which are thought to play a crucial role in the development of the majority of cancers. Here, we present a novel coarse-grained approach in modeling G-quadruplexes that accounts for their structural flexibility. We apply it to study the polymeric properties of G-quadruplex multimers, with and without crowder molecules, to mimic in vivo conditions. We find that, contrary to some suggestions found in the literature, long G-quadruplex multimers are rather flexible polymeric macromolecules, with a local persistence length comparable to monomer size, exhibiting a chain stiffness variation profile consistent with a real polymer in good solvent. Moreover, in a crowded environment (up to 10% volume fraction), we report that G-quadruplex multimers exhibit an increased propensity for coiling, with a corresponding decrease in the measured chain stiffness.

## Introduction

**Figure 1:**
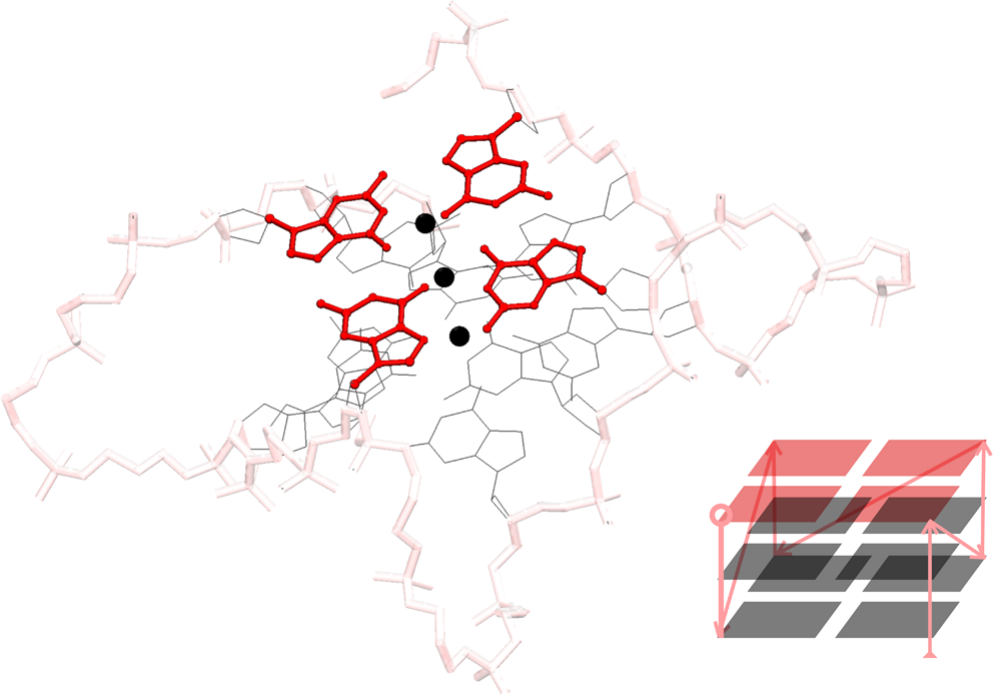
Stylized and simplified (lower right) structure of a folded conformation of the Tel22 sequence (Protein Data Bank entry 1KF1^4^), with the so-called, parallel topology. The gray wireframe outlines Guanine nucleotides (other nucleotides not shown). Four in-plane Guanine nucleotides linked by Hoogsteen hydrogen bonds (not shown) constitute a G-tetrad (highlighted in red). The scaffold (and lines in the simplified view) highlighted in pink outlines the G4 monomer (sugar–phosphate) backbone. Monovalent *K*+ ions are depicted as black spheres. Arrows in the simplified structure view indicate the strand polarity of the backbone.

G-quadruplexes (G4s) are noncanonical DNA conformations, formed by guanine-rich oligonucleotides. Structurally, a G4 consists of an array of quasi-planar tetrads of guanine tracts (G-tetrads). G4s are polymorphic structures^1−14^ with three main topologies^15−17^ (parallel, antiparallel, and hybrid), long folding time scales, and a range of long-living, quasi-stable topologies that commonly coexist in solution.^18−25^ Note that, while the references summarized in the previous statement refer mainly to telomeric G4, the characteristics described therein are not telomeric-G4-specific. An example of the structure of G4 is given in Figure 1. The morphology of G4s is largely achieved via a network of Hoogsteen-type hydrogen bonds, pi-stacking interactions, and coordinating cations,^26,27^ and is contingent on environmental factors such as the cation type and concentration, molecular crowding, and dehydration conditions.^28−33^ Sequences capable of forming G4s are abundant in the genomes of higher eukaryotes,^34−36^ and particularly concentrated in telomeric regions, constituting up to 25% of all DNA G4s.^27^ Biological role(s) of G4 DNA and its metabolizing enzymes (e.g., helicases) in DNA transcription and genomic stability are not fully understood. G4s have been observed in vivo,^37−40^ and are believed to play a role in regulating transcription, translation, DNA replication, RNA localization, and various other crucial biological functions.^41−43^ G4s have received considerable attention as targets for drug design.^44−47^ They have been shown to inhibit telomerase and HIV integrase.^48^ There is a potential for specific G4-stabilizing compounds to be utilized as anticancer or antiviral medications.^49−54^ Moreover, G4s have been extensively explored as promising building blocks in synthetic biology and nanotechnology.^55,56^

While much of the research on G4s has concentrated on their monomeric state, telomeric sequences have the potential to form higher-order multimeric structures with a variable number/arrangement of G4 units,^57^ with distinct biological roles and special interest as potential drug targets.^58,59^ The stacking interfaces between G4s could be viewed as binding grooves, valuable for drug targeting. Given that single-stranded telomeric overhang length ranges from 50 to ≈600 nucleotides,^60,61^ with a conservative estimate of the number of nucleotides needed for a G4 to form being ≈25, it is not surprising that G4 multimers form.^62^ G4 multimers tend to form in biological environments that are densely packed with various biomolecules.^63^ It has been reported that crowder molecules (CMs) tend to stabilize G4s and support the formation of multimers.^33,64,65^ However, how exactly they affect the dynamics of G4 formation is not well understood.

There is some disagreement about the telomeric G4 multimer formation in solution. Some literature reports G4 multimers that are describable as beads-on-a-string, with a maximal number of G4 for a given sequence.^66−70^ Others propose a more rigid backbone, where G4 multimers adopt compact, rod-like structures via stacking interactions.^71−73^ Some publications report highly flexible arrangements with large gaps occurring between G4s.^74−76^ Consequently, there is currently no clear view on the flexibility of G4 multimers. This is a crucial question to answer, as flexibility relates to the functions of biopolymers,^77−80^ and needs to be quantified in order to scrutinize any physical quantities that change according to the distance from the object of interest (i.e., counterion concentration and distribution for poly electrolyte chains).^81^ This is especially relevant for G4 multimers, as the complex interplay with crowding molecules and ligands could strongly affect multimer flexibility.^58,82,83^

There is little structural information on multimeric G4s, as X-ray crystallography and/or Nuclear Magnetic Resonance spectroscopy studies have been struggling to deal with longer nucleic acid sequences.^62^ Despite the recent advancements in small-angle X-ray scattering (SAXS) experiments, the interpretation of data necessitates the use of complex ab initio space-filling models or atomistic simulations.^84−103^ The extreme computational cost, system size, and time scale restrictions inherent to atomistic simulations limit their utility in the study of G4 multimers. Furthermore, strength of stacking interactions between G4s, which is crucial to determine G4 multimers’ conformation, is not well reproduced by current atomistic force fields.^104−106^ Coarse-grained (CG) simulations are a way to reach where atomistic simulations cannot be directly applied. Note that SAXS is not the only method for probing structural information on multimeric structures. Photon Correlation Spectroscopy and Dynamic Light Scattering can, in principle, also provide valuable insights into quadruplex dimensions and their behavior in solution.^107−111^

Recently, using hard cylinder Monte Carlo simulations, we enabled the direct interpretation of in vitro SAXS experiments on the self-assembly of Tel22 (d(TTAGGG)3)) and Tel72 (d(TTAGGG)12) multimers, with and without ligands (TMPyP4 porphyrin and BRACO-19, respectively).^112^ However, this approach cannot be used to scrutinize phenomenology where resolving the structural features of G4s is necessary (length scale less than a few nanometers). Here, we present a CG model of G4 mono- and multimers, validated against in vitro experimental data from Monsen et al.^84^ For an in-depth discussion of the experimental systems, we refer the reader to their exhaustive analysis. We perform long-time scale, bulk Molecular Dynamics^113^ (MD) simulations of G4 multimers, *M* × G4, where *M* denotes the number of monomers and *M* ∈ {1, 2, 3, 4, 10, 15, 20}. To the best of our knowledge, this is the first study to simulate long G4 multimers and the first to investigate their behavior in both crowded and uncrowded environments. Using a novel CG model, we characterize the polymeric properties of G4 multimers in a general way, within the framework of real polymer theory. In this work, we provide new insights, set expectations, and lay a theoretical foundation for future in vitro studies of long G4 multimers.

## Methodology

### Modeling Details

**Figure 2:**
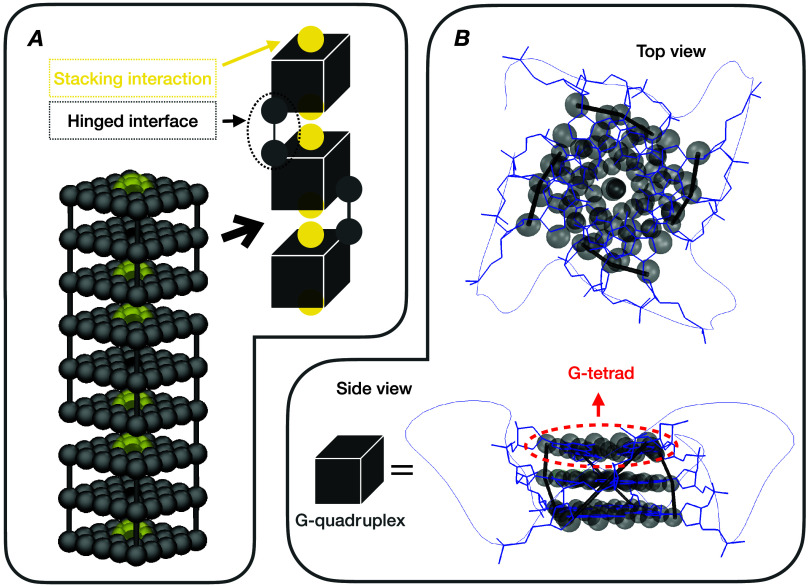
Panel A: Simulation render of a G4 trimer, consisting from permanently bonded G4 monomers, joined by pairwise hinged interfaces and stacking interactions. The CoM particles are colored black. Particles outlining a G-tetrad (see panel B) and the links between adjacent G-tetrads and/or the neighboring G4 monomers are colored gray. The central attraction between the CoM particles on the outer G-tetrads (i.e., stacking interaction between G4 monomers) are depicted as transparent yellow spheres. Relative sizes and distances correspond to the interaction minima. Panel B: Qualitative superposition of the CG model of a G4 monomer and a G4 monomer folded from a *Tel22* sequence (Protein Data Bank entry 1KF1^4^). Visualizations made using the VMD molecular visualization program.^114^

An annotated depiction of our CG model of a G4 multimer can be seen in Figure 2. The baseline structure in our simulations is the G-tetrad, modeled as a 5 × 5 grid of equidistant spheres (see Figure 2 Panel B). The excluded volume of a sphere with a characteristic diameter σ is realized via the Weeks–Chandler–Andersen (WCA) potential:^115^
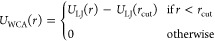
1where *U*_LJ_(*r*) is the conventional Lennard-Jones potential:
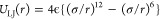
2where the cutoff value is *r*_cut_ = 2^1/6^σ. The parameter ϵ defines the interaction strength (relative to the energy scale). Only the center-of-mass (CoM) particle (black spheres in Figure 2 Panel A) in each G-tetrad is propagated using the equations of motion (eqs 4 and 5, respectively). The rest of the spherical particles outlining the G-tetrad are virtual (gray spheres in Figure 2), meaning that they have a fixed position with respect to the CoM particle, which incidentally is the only particle that carries mass. Note that the frictional coupling is set accordingly. The moment of inertia tensor of all CoM particles is modified to account for the halo of virtual sites outlining the G-tetrad shape.

A G4 monomer consists of three G-tetrads, linked together via finitely extensible, nonlinear elastic (FENE) bonds:^116^
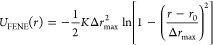
3where *K* is the rigidity of the bond, Δ*r*_max_ is the maximal stretching length and *r*_0_ is the equilibrium bond length. Specifically, the corner particles in adjacent G-tetrads are linked. Making multimeric structures out of G4 monomers is achieved by introducing FENE linkers between a randomly chosen pair of corner particles on adjacent G-tetrads of neighboring G4 monomers. In order to mimic the stacking interactions between monomers,^112^ the center-of-mass (CoM) particles of the outer G-tetrads exhibit a central attraction, realized via Lennard-Jones interaction potential. The Lennard-Jones interaction (eq 2), used to mimic the stacking interactions between monomers, is a good representation of the affinity monomers might have for the solvent and/or each other, and is often used in computational studies for this purpose.^117^ Tuning the stacking interaction is a simple but effective way to mimic the solvent in experiments, as long as one is exclusively interested in equilibrium properties. The CoM particles within the same G4 do not have a central attraction between them.

This model is designed to minimize complexity (i.e., the number of tunable parameters) and to enable scalable, efficient simulations of G4 systems. It also reflects a particular perspective on the structure of a G4. Consider a single telomeric G4 monomer, folded from an AG3(T2AG3)3^118^ or 2JSL sequence.^84^ In physiological conditions, such a monomer consists of three G-tetrads that contain two *K*+ or three *Na*+ stabilizing cations. In fact, most of the structural stability of a G4 monomer comes from the electrostatic interaction (in this context, the hydrogen bonds are also electrostatic interactions) between the G-tetrads and the ions within the G4.^66,70,72^ Given that the electrostatic interactions within the G4 are effectively short-ranged due to evident interaction screening, we take the view that a G-tetrad can be represented as a purely topological, steric hindrance, firmly coupled to a monovalent ion. Since the G-tetrads in a G4 monomer are linked via short but elastic liners, whereas the intermonomer links are comparable to the average intertetrad links, the overall structure is rather soft.

We validate our model and its corresponding parameter choices by comparing the simulated results with the experimental ones reported by Monsen et al.^84^ Specifically, we reference the experimental SAXS data for the 2JSL, Tel48, Tel72, and Tel96 sequences reported in their study. In our simulations, these sequences correspond to *M* × G4 multimers, where *M* ∈ {1, 2, 3, 4}, respectively.

### Simulation Method

We perform MD simulations using the ESPResSo software package.^119^ The carrier fluid was represented implicitly, via the Langevin thermostat at fixed temperature *T*.^113^ In practice, it means that the Langevin equations of motion are integrated over time *t* numerically:
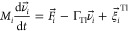
4
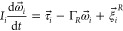
5where for the *i-*th particle in eq 4, *M*_*i*_ is, in general, a rank two mass tensor, that in our case of isotropic monomers reduces to a scalar, *F⃗*_*i*_ is the force acting on the particle, ν⃗_*i*_ denotes the translational velocity. Γ_Tl_ denotes the translational friction tensor that once again in our particular case reduces to one scalar friction coefficient. Finally, ξ⃗_*i*_^Tl^ is a stochastic force, modeling the thermal fluctuations of the implicit solvent. Similarly, in eq 5, *I*_*i*_ denotes *i*-th particle inertia tensor (scalar for a homogeneous sphere), τ⃗_*i*_ is torque acting on it, ω⃗_*i*_ is particle rotational velocity. As for the translation, Γ_*R*_ denotes the rotational friction tensor that reduces to a scalar for our monomers, and the ξ⃗_*i*_^*R*^ is a stochastic torque serving the same purpose as ξ⃗_*i*_^Tl^. Both stochastic terms satisfy the conditions on their time averages:^120^
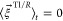
6
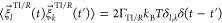
where *k*, *l* = *x*, *y*, *z*.

Forces and torques in eqs 4 and 5 are calculated from interparticle interaction potentials. Each simulation box contained 6000 G-tetrads, which combine into 6000/(3*M*) multimers. Simulations were performed at a fixed concentration of *C* = 0.6 mM without CMs or *C* = 5 mM with CMs. We used periodic boundary conditions and a cubic simulation box to approximate infinite systems and extract bulk properties at equilibrium. For the integration, the velocity Verlet algorithm was used,^121^ with a time step of 0.01 in simulation units (SU; see the Simulation Units and Mapping to Physical Parameters section for more details on the simulation units). In all cases, the initial configurations were generated so that both the positions and orientations of the largest predefined structures are appropriately randomized. We ensure that the system relaxes into an equilibrium configuration by running an integration cycle for 2 × 10^6^ integration steps. To obtain statistically significant results, we present averages over 100 uncorrelated data sets (10 simulation snapshots separated by 1 × 10^5^ integration steps, across 10 independent simulation runs). The snapshot separation was determined as the number of subsequent snapshots necessary for the position autocorrelation function to decay to zero. Based on this, we (randomly) subsampled our data to obtain uncorrelated data sets.

### Simulation Units and Mapping to Physical Parameters

In this subsection, we give a detailed overview of the units used in our simulations. We did not attempt to fit the parameters to match the scattering data for the specific experimental systems studied in Monsen et al.^84^ Instead, our interaction strength and parameter choices were informed by the parameter space explored in Rosi et al.,^112^ which studied different telomeric sequences, as a proof of the robustness of our parameter choices. The same parameters were used regardless of sequence/monomer number. We chose the time scale and length scale in our MD simulations to be [*t*] = 1 × 10^–9^ s and [*x*] = 0.4 nm, respectively. Note that the length scale corresponds to σ = 1 SU, which is the diameter of a single particle in the 5 × 5 grid of particles outlining a G-tetrad, in simulation units. The energy scale in the simulations is set to room temperature, *T* = 298.15 K, which corresponds to the Langevin thermostat temperature of *k*_B_*T* = 1 SU and steric repulsion strength ϵ_WCA_ = 1 SU. The central attraction strength has been set to ϵ_LJ_ = 5 SU. The above-stated parameter choices uniquely define a mass scale. It is, however, completely arbitrary as far as the scope of this work is concerned. The factor *K* of the potential in eq 3 is set to *K* = 10 SU. The equilibrium distance for FENE bonds is *r*_0_ = 2σ, and their maximum extension is Δ*r*_max_ = 1.5*r*_0_. With these parameters, the aspect ratio of a CG G4 monomer in our simulations (the ratio of the longest, i.e., principal, to the shortest component of the gyration tensor, given in eq 9) aligns with the experimental aspect ratio of a G4 monomer folded from a Tel22 sequence, as reported in Libera et al.^118^ The experimental aspect ratio was determined by fitting the form factor of the monomer to that of a cylinder (the ratio of the cylinder height to the diameter of its base).

## Results and Discussion

Studying the profiles from SAXS measurements is a powerful way to characterize the structure of biological macromolecules such as G4 multimers. In addition to the structural information, scattering intensities are a way to experimentally access the structure factor, and by proxy, the pair-correlation function of the system. The pair-correlation function captures thermodynamic information about a given system and can, for example, be used to calculate the expectation value of observables, or even write the equation of state of a given system.^122,123^ On the other hand, the structure factor can be calculated directly from simulated data using
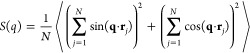
7where ***q*** is the scattering wavevector, *N* is the total number of particles and *r*_*i*_ is the position of the *i-*th particle. The crucial links are that the scattering intensity *I*(*q*) ∝ *S*(*q*) and that the *S*(*q*) is a Fourier transform of the pair-correlation function. So if an *S*(*q*) calculated from the simulated data of a CG model reproduces the experimental *I*(*q*) (up to a scaling factor), the CG model will reproduce the corresponding thermodynamic properties of the experimental system. Here, it is important to underline the implicit assumption that light-particle interaction effects present in experimental SAXS experiments, typically encompassed as a part of a measured form factor, do not warrant a special treatment here. In the low to intermediate *q* range, the *I*(*q*) is dominated by the pure *S*(*q*) signal, whereas, in the high-*q* range, the *I*(*q*) signal is noisy and does not convey useful information. Therefore, comparing experimental *I*(*q*) data with simulated *S*(*q*) is justified and can be used to validate the model. This is also why from this point onward, we refer to simulated *S*(*q*) as a simulated scattering intensity (in other words, from this point on, *I*(*q*) and *S*(*q*) are treated as equivalent). Scattering intensities from simulations were calculated using the *espressoSq* library.^124^ For more detail, see the Supporting Information.

**Figure 3:**
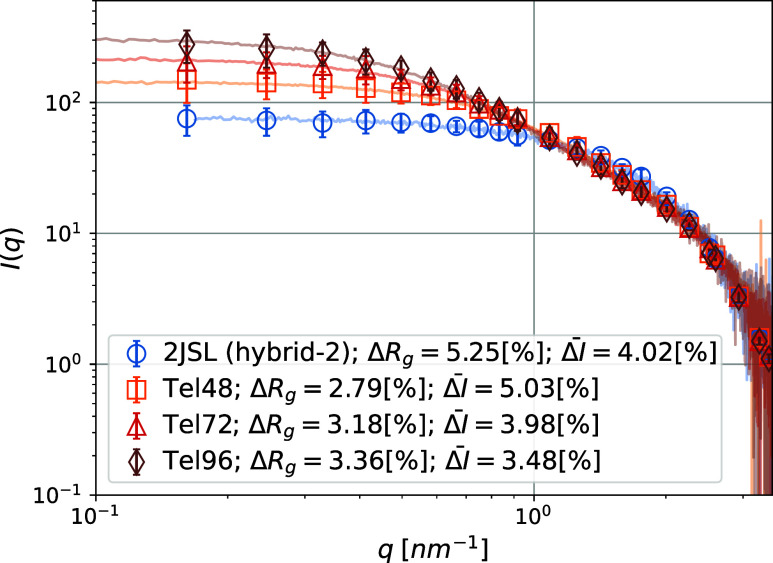
Comparison of scattering intensity *I*(*q*) between in vitro G4 multimers from Monsen et al.^84^ and CG simulations. The color coding, the relative error in *R*_g_ from Guinier analysis, Δ*R*_g_ (see Supporting Information for details about the fitting procedure and parameters) and the mean relative error between experimental and simulated I(q),  are shown in the legend. Error bars represent the standard deviation of simulated data.

In Figure 3, we are superimposing experimental and simulated *I*(*q*), denoted as *I*_exp_(*q*) and *I*_sim_(*q*), respectively, where one can see that our CG model captures the experimentally measured SAXS profiles very well, with a mean relative error  for all sequences. This validates the CG model and positions it as a viable tool to study the equilibrium properties of G4 multimers. Here, it is important to note that, while the parameter set we used is not unique, it is fairly robust and by no means arbitrary. For example, if the strength of the stacking interaction relative to thermal fluctuations were too high, the *I*(*q*) would be overestimated in the low-*q* region (see Rosi et al.^112^). Moreover, if the flexibility of a G4 monomer were not captured correctly, or if the design of the (short) hinged interface were not representative of the experimental system, the intermediate-*q* curvature and/or slope would be incorrect. Similarly, if our model did not capture the dimensions and/or G4s, we would see features contradicting the experimental data in the high-*q* range.

In Monsen et al.,^84^ it is stated that the studied sequences fold into multimers with the maximum possible G4 monomer number. We assume that, for a given sequence, the corresponding CG G4 multimer has a fixed G4 monomer number, equal to the maximum possible G4 monomer number for that sequence. Furthermore, all G4 multimers in a given simulation are assumed to have the same G4 monomer number. Therefore, our data corroborates that differences in the curvature of the intermediate- to high-*q* range can be entirely attributed to increasing monomer numbers across the samples.

Experimental *I*(*q*) data on polymer-like structures is typically used to extract polymeric properties of the systems studied, such as the radius of gyration *R*_g_.^125^ Formally, the (mass-independent) radius of gyration is defined as
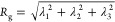
8where λ_1_ > λ_2_ > λ_3_ are the eigenvalues of the gyration tensor:
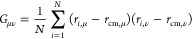
9where *r*_*i*,μ_ and *r*_cm,μ_ are the μth Cartesian components of the position of the *i-*th particle and the center of mass, respectively. The summation is carried over all *N* particles. *R*_g_ can be extracted from scattering experiments using the well-known Guinier’s approximation.^126^ Namely, in the low-*q* range, the scattering intensity *I*(*q*) can be approximated as
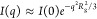
10We calculated the *R*_g_ using the Guinier approach for both simulated and experimental data (*R*_g_^sim^ and *R*_g_^exp^, respectively), finding a very good match with a relative error Δ*R*_g_ = |*R*_g_^sim^ – *R*_g_^exp^|/*R*_g_^exp^ ≲ 5%. The Guinier approach relies on various assumptions about the experimental system and involves rather sensitive fitting. On the other hand, eq 8 can be used directly on the simulated data, which is, in our opinion, a transparent and preferable method to measure *R*_*g*_ for the experimental system since our model fits the entire *I*(*q*), instead of just the Guinier region, which is a fraction of scattering data. To qualify this distinction, we define the relative error δ*R_g_* = |*R*_g_^guinier^ – *R*_g_^direct^|/*R*_g_^direct^ to estimate the difference in simulated *R*_g_ if estimated from Guinier analysis (*R*_g_^guinier^, which is equivalent to *R*_g_^sim^ used above) or using eq 8 (*R*_g_^direct^). As can be seen in Table 1, δ*R*_g_ indicates that there can be up to a 8% discrepancy in measured *R*_g_ depending on the measurement approach. This highlights the role of scalable, CG models, such as the one presented here, as tools where a reduced set of fit parameters (reduced complexity) can be tuned to match experimental measurements and, through that, enable further insights.

**Table 1:** Percent Difference in Measured Radius of Gyration, δ*R*_g_, Based on Measurement Approach, Where We Used Either Guinier Analysis or Direct Analysis UsingEq 8

	1 × G4	2 × G4	3 × G4	4 × G4
δ*R*_g_ [%]	2.9377	4.1977	8.5696	6.0594

**Figure 4:**
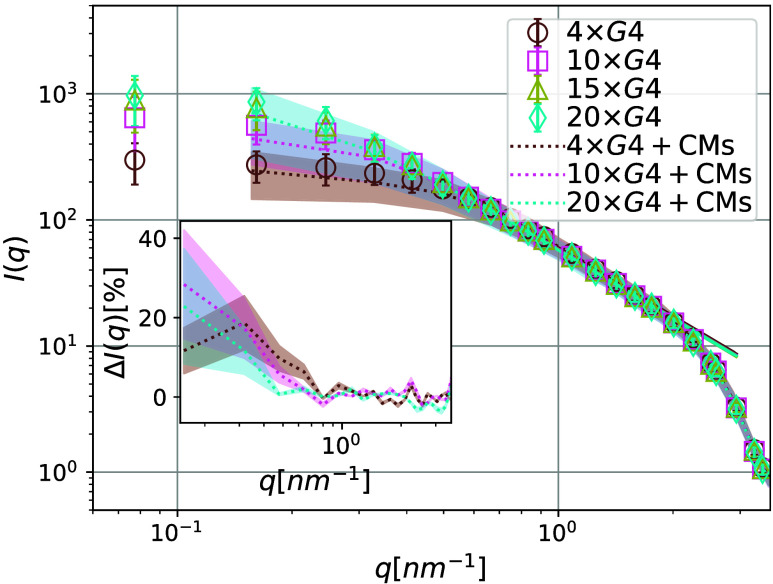
Simulated scattering intensities *I*(*q*) for *M* × *G*4 multimers where *M* ∈ {4, 10, 15, 20}. The intermediate-*q* slope was fitted using *f*(*q*) = *a* + *bq*, with *b* = – 1.82 ± 0.01. Data without CMs are shown with squares, with *C* = 0.6 mM. Simulations with CMs (*C* = 5 mM) are shown with dotted lines, interpolated for clarity. The inset shows the percentage difference in scattering intensity, Δ*I*(*q*), between simulations with and without CMs, with linear-log axes. Color coding is explained in the legend. Color-matched halos and error bars represent standard deviation for the data with and without CMs, respectively.

Having established the validity of our CG model, we proceed to study long G4 multimers, which are beyond the scope of current experimental and/or atomistic simulation studies. In Figure 4, we show simulated *I*(*q*) profiles for long *M* × G4 multimers, where *M* ∈ {4, 10, 15, 20}. Looking at Figure 4 one notes the formation of two linear regions, in the low and the intermediate-*q* range, respectively. In the low-*q* range, we see the asymptotic approach to a maximal *I*(*q*) height with increasing G4 monomer number. Furthermore, the scattering profiles for 15 × G4 and 20 × G4 multimers do not approach the *y*-axis completely horizontally in the low-*q* range, which signals interparticle interactions and repulsion.^125^ Both of these points are consistent with an image of a flexible, coiling polymer. The slope of the *I*(*q*) linear region in the intermediate-*q* range can be related with the distribution of bond vectors in a polymeric sample, where it is known that a slope of −2 corresponds to ideal polymer statistics.^127,128^ The slope we extract, however, hints that there are nontrivial intermonomer correlations along the polymeric backbone.

To study the effect of excluded volume in a crowded environment on the properties of G4 multimers, we simulated *M* × G4 multimers where *M* ∈ {4, 10, 15, 20} at 1 and 10% volume fractions of CMs. The CMs are represented as WCA spheres with diameter σ_crowder_ = 6 SU; for comparison, a sphere circumscribed around a single G4 monomer would have a diameter of approximately 8.5σ. This simulation setup is designed to set expectations for in vitro studies of crowded G4 multimers in a good solvent, where, for example, long PEG molecules are typically used.^64^ However, it is important to note that we take the view that CMs are, by definition, inert to the species of interest (G4 multimers) and exhibit only excluded volume interactions. Taking PEG as an example, while it is considered biologically inert, it has been reported not to act as a pure crowding agent and has been described as a poor mimic of the intranuclear environment.^129^ Therefore, one must keep in mind that the term CM is often used for a broader set of molecules than would fit the aforementioned definition. The underlying assumption in such studies—that the most prominent effect of typical molecules used in vitro to mimic crowded environments is steric—is reasonable. However, it is important to remember that CMs in this broader sense can also bind to G4, form complexes with the monovalent ions stabilizing G4, and significantly affect the folding/unfolding dynamics of the sequences. Moreover, different in vitro-used CMs can facilitate the formation of particular G4 conformations. Thus, if one deviates from the strict definition of what constitutes a CM presented above, one should account for electrostatic interactions and, more generally, consider that sequences capable of forming G4 behave as polyelectrolytes.

Looking at Figure 4, where we also provide the scattering profiles for 4 × G4, 10 × G4 and 20 × G4 multimers with the CMs at volume fraction ϕ = 10%, we can see that the presence of crowding molecules reduces the *I*(*q*) in the low-*q* region, with a correspondingly increased variance. As seen in the inset of Figure 4, the noted reduction in *I*(*q*) is statistically significant and can be attributed to the increased coiling propensity of G4 multimers in a crowded environment. We expect this effect would be enhanced in human cells – where the volume fraction of CMs is estimated to be around 30–40%.^130^

The in vitro G4 multimers discussed in Monsen et al.^84^ are reported as semiflexible polymers, consistent with rigid G4 units linked by hinged interfaces. Similar reports (and contradictory ones) can be found across the literature summarized above, where *R*_g_ as a function of monomer numbers is fitted with a random Gaussian coil and/or the Worm-like chain model to estimate persistence length *L*_p_. These models are known to reproduce the stiffness of canonical duplex DNA.^131^ While such an analysis is certainly useful, it is not sufficient to characterize the flexibility of G4 long multimers. Flexibility of macromolecules is commonly characterized using the notion of persistence length.^132^ Classically, *L*_p_^id^ is calculated from the decay of the autocorrelation function between vectors *a⃗*_*k*_ connecting each pair *k* of neighboring monomers along the backbone, separated by *N*_b_ bond vectors:
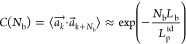
11where a bond vector is defined as the center-of-mass distance between a pair of adjacent monomers and *L*_b_ is the average bond vector length. For real polymers, this is not the case, as nontrivial excluded volume correlations persist throughout the polymeric backbone, and exhibit a power law decorrelation profile.^133^ More generally, persistence length is a chain property that can, within real polymer theory, vary substantially along the chain backbone. Schäfer and Elsner^134^ have shown that, to a very good approximation:
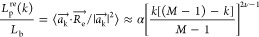
12where *R*_e_ is the end-to-end distance vector.

**Figure 5:**
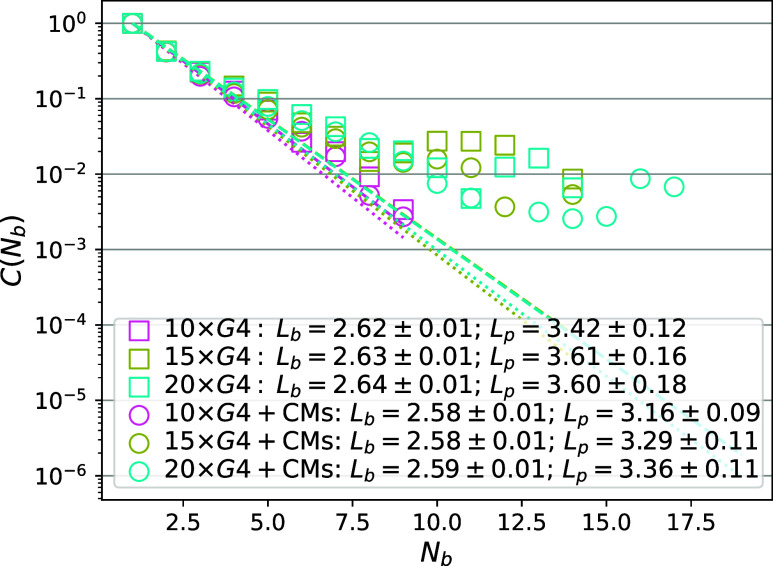
Bond correlation function *C*(*N*_*b*_). Data with (10% volume fraction) and without CMs is shown with circles and squares, respectively. Fits of eq 11 are shown as dash-dotted (with CMs) and dashed lines (without CMs). The color coding and the extracted *L*_*p*_ values (*L*_p_^id^ in eq 11, given in nm) are provided in the legend. The *y*-axis is logarithmic.

**Figure 6:**
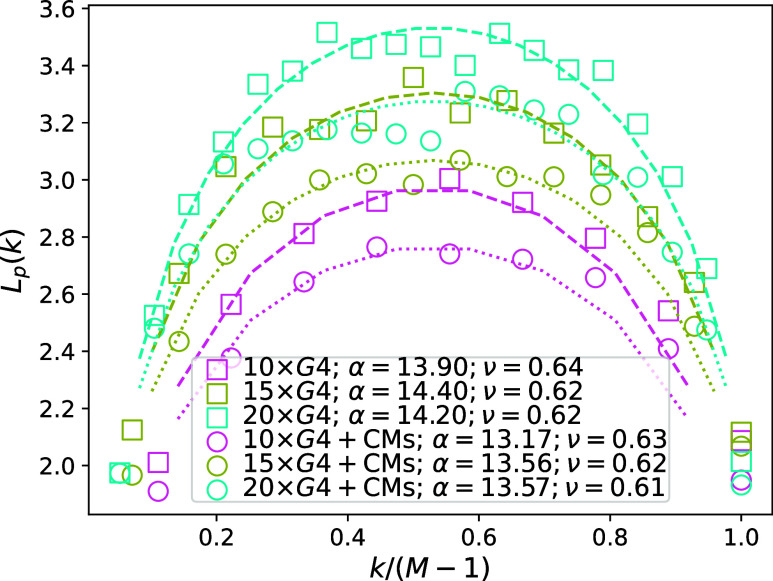
Persistence length *L*_p_ (*L*_p_^re^ in eq 12, given in nm). Data without CMs are shown with squares, and with 10% CM volume fraction with circles. Fits of eq 12 are shown as dashed (no CMs) and dotted lines (CMs), with color coding in the legend.

Looking at Figure 5, we can see how well the classic notion *L*_p_^id^ can be applied in the context of G4 multimers. The *C*(*N*_b_) corresponds to the expected exponential decay only for 10 × G4 multimers. For longer multimers (15 × G4 and 20 × G4), we observe the onset of a power law decay, characteristic of real polymers. It is important to note that the *M* × G4 multimers we studied are short from the perspective of polymer physics scaling theories.^135^ Since deviations start being notable only for small correlations *C*(*N*_b_) ≈ 0.05, we can use *L*_p_^id^ as a monomer number independent estimate of the stiffness of G4 multimers. We obtain *L*_*p*_^id^ = 3.54 ± 0.15 nm, which is compatible with the values reported by Monsen et al.^84^ However, G4 multimers do not follow ideal polymer statistics. While *L*_p_^id^ is a useful *relative* quantity,^136,137^ it is not strictly correct to apply it to G4 multimers. Looking at Figure 6, we can see the *L*_p_^re^ fits to our simulated data on 10 × G4, 15 × G4 and 20 × G4 well. This elucidates key properties to be expected from long G4 multimers, which is that, as is characteristic of real polymers, chain stiffness varies within a G4 multimer, well captured by the concave shape of eq 12. The *L*_p_^re^ and *L*_p_^id^ (monomer number independent) values we measured are compatible. Consistent with what we have observed in Figure 4, the presence of CMs systematically decreases the *L*_p_. Obtaining a measure of *L*_p_^re^ that is *M* independent is not feasible as it is necessary to consider much larger monomer numbers to make such an estimate sensible.^133^ The matter is further complicated by the fact that it is highly unlikely for G4 multimers with a higher monomer number than we have studied here to form.^61,138^ Having said that, the analysis we present here is sufficient to show that, in the in vitro conditions reported in Monsen et al.,^84^ the scaling exponent is close to the expected value for a real polymer in a good solvent.^132^ Our results support a view where stacking interactions between the monomers in a G4 multimer are weak. In this case, provided the short hinged interfaces between the monomers, it is clear that G4 monomers bend and twist away from each other to maximize entropy, in which case the steric hindrance coming from the monomer shape is not relevant. Preventing neighboring G4 monomers to twist away from each other will require significant solvent induced hydrophobic interactions, at which point G4 multimers would probably also start to aggregate.

## Conclusions

One of the most distinctive features of G4s is the quasi-cubic monomer shape. Even for soft systems, monomer shape reflects on to polymeric properties substantially, provided that both the translational and rotational degrees of freedom between the monomers are coupled and the average intermonomer distance is low.^139^ This is not the case for G4 multimers, which can exhibit polymeric properties in line with a flexible real polymer in a good solvent, at least as far as we can see from in vitro studies. It is interesting to consider that the fact that G4 multimers sit between single-strand DNA and duplex DNA in terms of flexibility,^62^ serves a functional purpose. It has been shown that ligands such as TMPyP4 porphyrin, broadly speaking, stack between G4 units^118^ (yellow terminals in Figure 2) and their action can be represented as an effective increase in stacking interaction strength.^112^ Moreover, this selective action provides a significant advantage in the use of G4 stabilizers as anticancer drugs.^140,141^ In light of the results we presented here it is clear that such ligands increase the stiffness of G4 structures, that are otherwise entirely flexible, especially in a crowded complex biological environment. Therefore, we suggest that the efficacy of anticancer G4 targeting ligands is closely related to the G4 multimer stiffness increase they cause. Hopefully, this work inspires further experimental studies to scrutinize this point and to further use CG models to study G4 systems, unlocking a variety of implicit and explicit solvent simulation studies that were previously not feasible. The CG model we present here specifically, can be used to investigate dynamics of G4 systems, which is something we are currently working on. In this respect, this model could be expanded to efficiently study the folding/unfolding pathways and aggregation kinetics of ligands and G4s.

## Supporting Information Available

Guinier analysis procedure; fitting parameters from the Guinier analysis are provided; radius of gyration extraction procedure and raw values are provided; details about structure factor calculation and technical detail are provided (PDF)
