# Supplementary material for: Polymeric Properties of Telomeric G-Quadruplex Multimers: Effects of Chemically Inert Crowders
Source: Biomacromolecules. 2025 Apr 8;26(5):3128–38. doi: 10.1021/acs.biomac.5c00176 (PMC12076513; doi:10.1021/acs.biomac.5c00176)
Supplement: Supplementary file 1 — bm5c00176_si_001.pdf [file bm5c00176_si_001.pdf]

Supplementary Information

Polymeric Properties of Telomeric G-Quadruplex Multimers: Effects of Chemically Inert Crowders

Deniz Mostarac,[*,†] Mattia Trapella,[‡] Luca Bertini,[‡] Lucia Comez,[¶] Alessandro Paciaroni,[‡] and Cristiano De Michele[†]

†*Department of Physics, University of Rome La Sapienza, 00185 Rome, Italy*
‡*Department of Physics and Geology, University of Perugia, 06123 Perugia, Italy*
¶*CNR - Istituto Officina dei Materiali (IOM), 06123 Perugia, Italy*

E-mail: deniz.mostarac@uniroma1.it

Guinier analysis

Our code iteratively adjusts the q_{min} and q_{max} (Guinier fit region) so that the coefficient of determination R^2 is maximized, with the added constraint that $q_{max} < 1.3$ (this limit was also used in Monsen et al.[1]). R_g is extracted via the standard procedure where the scattering intensity is considered as a function of $(qR_g)^2$. In the Guinier region, the scattering intensity should appear as a linear function on a log-linear plot. Therefore, we can extract both the R_g and R^2 parameters from linear regression in the appropriate range. Final fit parameters are provided in Tables 1 and 2, for simulated and experimental data, respectively. Note that we did not employ an optimization strategy when superimposing experimental and simulated scattering intensities. Instead, we simply used two scaling factors, one for q and one for $I(q)$, respectively. The q-axis scaling can be estimated directly from the units. The $I(q)$-axis scaling factor was determined by fitting the high-q (noisy) region of the experimental data such that its mean value is zero. In this way, we could superpose the experimental scattering intensities for different multimers, and fit the simulated $I(q)$ naturally. It is in principle possible to better superimpose the experimental and simulated data by determining the scaling coefficient by minimizing the mean relative error or a similar error estimator. This was, however, not necessary to draw the conclusions presented in this work.

Structure factor

As given in the main text, the structure factor can be calculated as:

$$S(q) = \frac{1}{N}\left\langle \left(\sum_{j=1}^{N} \sin(\mathbf{q}\cdot\mathbf{r}_j)\right)^2 + \left(\sum_{j=1}^{N} \cos(\mathbf{q}\cdot\mathbf{r}_j)\right)^2 \right\rangle,$$

where q is the scattering wave vector, N is the total number of particles, and r_i is the position of the i-th particle. It is worth taking some time to discuss what goes into calculating this quantity from simulations and some of the optimizations that make the calculation feasible for large systems. The optimizations discussed here are a part of our open-source structure factor calculation library *espressoSq*,[2] which was used to calculate simulation structure factors in this work. In a finite simulation box, there is a finite number of q-vectors where $2\pi/L \leq |q| \leq 2\pi/L$. In practice, however, one can specify an additional order of calculation parameter, which determines the resolution of the calculation. In this case, $|q = 2\pi/(L\sqrt(i))$ where i is an integer going from 0 to the square of the order parameter. Furthermore, as i in-

Table 1: Guinier fit parameters on simulated scattering intensity profiles. Simulation units are denoted with SU.

	$1 \times G4$	$2 \times G4$	$3 \times G4$	$4 \times G4$
q_{min} [nm^{-1}]	0.1759	0.1331	0.1640	0.2016
q_{max} [nm^{-1}]	1.2730	1.2103	1.2507	1.2769
R_g [SU]	2.4449	4.2116	5.0006	6.1718
R^2	0.9640	0.9830	0.9933	0.9891

Table 2: Guinier fit parameters on the experimental scattering intensity data from Monsen et al.[1]. Simulation units are denoted with SU.

	$2JSL$	$TEL48$	$TEL72$	$TEL96$
q_{min} [nm^{-1}]	0.1738	0.1260	0.1734	0.2143
q_{max} [nm^{-1}]	1.2879	1.1837	1.2791	1.2948
R_g [SU]	2.5805	3.9858	5.2875	6.5606
R^2	0.9734	0.9943	0.9962	0.9946

creases, so does the number of possible k, l, m indices defining the degenerate q-vectors for a given $|q|$. Without an optimization strategy, calculating the $S(q)$ for bulk simulations can be prohibitively slow. The most obvious optimization would be not to perform the calculation for every $|q|$. A convenient approach is to choose a $|q|$ subset such that the $|q|$-axis point spacing is uniform on a logarithmic scale. Furthermore, for a given $|q|$, there can be a large number of corresponding q-vectors. One can set an upper limit on the number of q-vectors to consider for each $|q|$, and subsample the set of possible q-vectors. One only needs to ensure that the orientations of the vectors in the sub-sampled set are uniformly distributed on the surface of a sphere, and that the sub-sampled set is sufficiently large. In this work, we used calculation order 60, and a maximal size of the sub-sampled q-vector set per $|q|$ of 300.

espressoSq library incorporates several technical optimizations. We implemented low-level code to leverage SIMD units on x86/arm CPUs (using AVX2/NEON ISA extensions, respectively) and parallelized the particle loop using OpenMP threading. Data parallelism is achieved using the standard MPI interface.

References

(1) Monsen, R. C.; Chakravarthy, S.; Dean, W. L.; Chaires, J. B.; Trent, J. O. The solution structures of higher-order human telomere G-quadruplex multimers. *Nucleic Acids Research* **2021**, *49*, 1749–1768.

(2) Mostarac, D. espressoSq. `https://github.com/stekajack/espressoSq`, 2024; Accessed: 2024-08-06 (YYYY-MM-DD).
